# Large-Scale Gene Expression Signatures Reveal a Microbicidal Pattern of Activation in *Mycobacterium leprae*-Infected Monocyte-Derived Macrophages With Low Multiplicity of Infection

**DOI:** 10.3389/fimmu.2021.647832

**Published:** 2021-04-16

**Authors:** Thyago Leal-Calvo, Bruna Leticia Martins, Daniele Ferreira Bertoluci, Patricia Sammarco Rosa, Rodrigo Mendes de Camargo, Giovanna Vale Germano, Vania Nieto Brito de Souza, Ana Carla Pereira Latini, Milton Ozório Moraes

**Affiliations:** ^1^ Laboratório de Hanseníase, Instituto Oswaldo Cruz, FIOCRUZ, Rio de Janeiro, Brazil; ^2^ Divisão de Pesquisa e Ensino, Instituto Lauro de Souza Lima, Bauru, Brazil; ^3^ Departamento de Doenças Tropicais, Faculdade de Medicina de Botucatu, Universidade Estadual Paulista, Botucatu, Brazil

**Keywords:** macrophages, *Mycobacterium leprae*, eQTLs, SNPs, host-directed therapy, leprosy, tuberculosis

## Abstract

Leprosy is a disease with a clinical spectrum of presentations that is also manifested in diverse histological features. At one pole, lepromatous lesions (L-pole) have phagocytic foamy macrophages heavily parasitized with freely multiplying intracellular *Mycobacterium leprae*. At the other pole, the presence of epithelioid giant cells and granulomatous formation in tuberculoid lesions (T-pole) lead to the control of *M. leprae* replication and the containment of its spread. The mechanism that triggers this polarization is unknown, but macrophages are central in this process. Over the past few years, leprosy has been studied using large scale techniques to shed light on the basic pathways that, upon infection, rewire the host cellular metabolism and gene expression. *M. leprae* is particularly peculiar as it invades Schwann cells in the nerves, reprogramming their gene expression leading to a stem-like cell phenotype. This modulatory behavior exerted by *M. leprae* is also observed in skin macrophages. Here, we used live *M. leprae* to infect (10:1 multiplicity of infection) monocyte-derived macrophages (MDMs) for 48 h and analyzed the whole gene expression profile using microarrays. In this model, we observe an intense upregulation of genes consistent with a cellular immune response, with enriched pathways including peptide and protein secretion, leukocyte activation, inflammation, and cellular divalent inorganic cation homeostasis. Among the most differentially expressed genes (DEGs) are *CCL5/RANTES* and *CYP27B1*, and several members of the metallothionein and metalloproteinase families. This is consistent with a proinflammatory state that would resemble macrophage rewiring toward granulomatous formation observed at the T-pole. Furthermore, a comparison with a dataset retrieved from the Gene Expression Omnibus of *M. leprae*-infected Schwann cells (MOI 100:1) showed that the patterns among the DEGs are highly distinct, as the Schwann cells under these conditions had a scavenging and phagocytic gene profile similar to M2-like macrophages, with enriched pathways rearrangements in the cytoskeleton, lipid and cholesterol metabolism and upregulated genes including *MVK*, *MSMO1*, and *LACC1*/*FAMIN*. In summary, macrophages may have a central role in defining the paradigmatic cellular (T-pole) vs. humoral (L-pole) responses and it is likely that the multiplicity of infection and genetic polymorphisms in key genes are gearing this polarization.

## Introduction

Schwann cells in the peripheral nerves and macrophages in the skin are the major host cells for *Mycobacterium leprae* (ML) infection (1). These cells operate with high plasticity, induced by different environmental factors, and *M. leprae* has a unique ability to subvert and reprogram these host cells in order to establish a more favorable niche in which to replicate and spread. Huge transcriptomic variations may induce phenotypic modifications, as evidenced by transformations in Schwann to mesenchymal-like cells upon infection (2). Furthermore, increased glucose uptake, mitochondrial shutdown, and lipid biosynthesis resembling the Warburg effect are all phenomena induced upon *M. leprae* infection in these cells, although some of these are restricted to specific clinical forms (3).

Clinical presentation of leprosy is a spectrum encompassing a myriad of manifestations (1), where the tuberculoid pole (T-pole) is restrictive to bacillus growth leading to localized disease, while the lepromatous forms (L-pole) present a permissive and disseminated clinical form with high bacterial loads (1). *M. leprae* has suffered a reductive evolution resulting in low genetic variability, which suggests that the diversity of the disease phenotypes is attributable to the host responses (4). This landscape makes leprosy a unique model to understand the mechanisms involved in the immunopathogenesis of infectious diseases.

In the skin, macrophages are pivotal in the host-pathogen interaction, having important roles from proinflammatory and microbicidal activity to tissue remodeling and wound healing, which are features of the so-called M1 and M2 macrophages, respectively. Most of the skin macrophages are derived from monocytes that migrate and differentiate under inflammatory stimuli, referred to as monocyte-derived macrophages (MDMs) (5). Macrophages present huge functional plasticity according to the milieu in order to maintain skin homeostasis (6). Although less efficient in T cell activation than dendritic cells, they are vastly superior in their phagocytic ability (7). Additionally, monocytes engulf the bacillus through phagocytosis and produce cytokines helping to dictate the host-specific immune response at the lesion. The initial immune events involved in leprosy disease progression are probably triggered by the macrophage-*M. leprae* interaction. This hypothesis can be reinforced by the fact that key innate immune genes, pattern recognition receptors, and autophagic genes have been associated with disease outcome in the mouse model for bacilli replication (infected footpads of Balb/C lineage mice) that carries the *NRAMP1* polymorphism. Furthermore, human genome-wide association studies and other genetic analysis has identified *PRKN*, *LRRK2*, *NOD2*, *TLR1*, and *MRC1* as genes associated with disease outcome and are expressed in *M. leprae*-infected macrophages (8). In this regard, polarized macrophages are found according to the clinical form of leprosy. In tuberculoid lesions, there is a predominance of classically-activated macrophages, which are able to partially contain *M. leprae* replication by activation of cellular responses, vitamin D-dependent pathways, and granuloma formation consistent with the M1 profile (9). On the other hand, lepromatous lesions present permissive, scavenging, phagocytic, and foamy macrophages with anti-inflammatory profile harboring a large number of bacilli, associated with a poor microbicide activity, which are the phenotypes of M2 macrophages (9, 10).

Pathways such as apoptosis and autophagy, combined with lower levels of proinflammatory cytokines and antigen presentation molecules are inhibited by live, but not dead, *M. leprae* in macrophages and Schwann cells (11–13). This type of response is triggered by type I interferons (IFNs) through a STING/TBK/IRF3 pathway that inhibits IFN-gamma and other microbicidal mechanisms within infected macrophages *in vitro* (12).

In a recent re-analysis of leprosy public microarray datasets, mainly comparing skin lesions from different clinical forms, we confirmed previous genes and pathways corroborating the predominance of cellular immunity, leukocyte differentiation, vitamin D receptor (VDR)-mediated microbicidal responses, and granuloma formation at the T-pole, while the L-pole exhibited scavenger receptors and lipid metabolism genes (14). Nevertheless, we also pointed out new differentially expressed genes (DEGs) in leprosy related to skin development and keratinocyte differentiation (14).

Macrophages play a central role in orchestrating gene modulation within the nerve or skin microenvironment. Nevertheless, genome-wide expression patterns from *M. leprae*-infected macrophages have never been obtained. The gene expression signatures of these macrophages can provide target discoveries for therapies and prevention strategies to interfere with leprosy outcomes. Herein, we show the DEGs of macrophages related to immune and other responses after *M. leprae* infection. Our data indicate that *M. leprae*-infected MDMs submitted to a low multiplicity of infection (10:1) for 48 h overly express a cellular immunity profile. This suggests an *M. leprae*-induced program that might create a granuloma formation type of response in the tissues. Moreover, a comparison between the dataset generated in this study with that of a previously published dataset involving *M. leprae*-infected primary Schwann cells *in vitro* was performed. Through this comparison it was found that the expression profile obtained after a higher multiplicity of infection (100:1) for 48 h has a distinct activation pattern, with genes associated with lipid biosynthesis and consistent with a scavenging and phagocytic profile. Understanding the interaction between macrophage and *M. leprae* is highly relevant in deciphering key features that could further aid in the definition of the clinical forms.

## Materials and Methods

### Study Subjects

Volunteers were recruited from the staff of the Lauro de Souza Lima Institute (Secretary of Health of São Paulo State, Brazil) including health care, cleaning, and security workers, as well as students. Firstly, 29 healthy individuals (14 men and 15 women, from 20 to 30 years), free from cancer and infectious and autoimmune diseases, were enrolled for the collection of peripheral blood. A signed, written informed consent was obtained from all participants. The study was approved by the Local Ethics Committee (Protocol: 56169616.5.0000.5475).

### Monocyte-Derived Macrophage (MDM) Differentiation

Fifty milliliters of peripheral blood were collected by venipuncture in tubes with anticoagulant (heparin). Peripheral blood mononuclear cells (PBMCs) were purified by density gradient (Histopaque 1077, Sigma Co., St. Louis, MO, USA) and monocytes were isolated by positive selection employing anti-CD14-coated magnetic microbeads (Miltenyi Biotec, Auburn, CA, USA) according to the manufacturer’s instructions. Purity greater than 95% was confirmed by flow cytometry using an anti-CD14 antibody (BD Biosciences, Franklin Lakes, NJ, USA).

Macrophages were differentiated by adherence, without using recombinant cytokines, in order to obtain a non-polarized macrophage with M0 phenotype, according described by Vogel and collaborators (15) with modifications. Briefly, monocytes were cultured in 24-well plates (5 × 10^5^ cells/well) in Iscove’s modified Dulbecco’s medium (IMDM, Gibco, Grand Island, NY, USA) supplemented with 10% of pooled human sera from the participants and 100 units/mL of penicillin and 100 μg/mL of streptomycin (Gibco; Thermo Fisher Scientific, Inc.) for 6 days at 37°C in 5% CO_2_ humidified atmosphere for the differentiation into macrophages. Cultures were fed by replacing half part of the complete medium on the third day. The percentage of differentiated macrophages was evaluated during the standardization of the protocols considering the expression of CD68 and was greater than 95%.

### *M. leprae* Purification and Macrophages Infection

*M. leprae* (Thai-53 strain, kindly provided by Dr. Yuji Yamamoto, NIH, Japan) was obtained from footpads of athymic nude mice, according to a previously described protocol (16). Briefly, each footpad was inoculated with 3 × 10^6^ acid-fast bacilli in 30 μL of saline solution. After 6-7 months, mice were euthanized and the footpads were collected (CEUA 06/2006). The protocol for bacilli recovery included footpad dissection, tissue isolation, and enzymatic digestion with 0.05% trypsin, followed by purification, quantification, and evaluation of ML viability using the Live/Dead BacLight Bacterial Viability Kit (Molecular Probes, Inc., Eugene, OR, USA). The *M. leprae* count was done after Ziehl-Neelsen staining (16, 17).

Previously differentiated MDMs were infected with *M. leprae* in the multiplicity of infection of 10 bacilli/cell (MOI = 10:1) and kept at 37°C in 5% CO_2_ humidified atmosphere. After 48 h of infection, macrophages were collected in TRIzol Reagent (Thermo Fisher Scientific, Carlsbad, CA, USA). Uninfected MDMs incubated under the same conditions were used as controls.

### RNA Isolation and Microarray

Total RNA was extracted by an in-house method using phenol:chloroform and isopropyl alcohol (18). Glycogen (Thermo Fisher Scientific) was added to improve RNA recovery. After centrifugation at 12,000 RPM for 5 minutes, the RNA pellet was washed with 70% ethanol, air dried, and resuspended in DEPC-treated water. RNA quality was evaluated on a Bioanalyzer 2100 Instrument (Agilent Technologies Inc., Palo Alto, CA, USA), by using the RNA 6000 Nano Kit (Agilent Technologies). For all samples, the RNA integrity number (RIN) was higher than 8.

For the transcriptome, RNA samples were then purified with the RNeasy MinElute Cleanup Kit (QIAGEN, Hilden, Germany). The reverse transcription synthesis and biotin labeling were performed with Epicentre TargetAmp Kit (Illumina, CA, USA), and transcriptomes were obtained by chip hybridization using the HumanHT-12 v4 BeadChip followed by scanning in iScan equipment (Illumina) according to manufacturer’s instructions.

### Microarray Data Analysis

Raw.idat files were imported into the R v. 3.6.1 (BiocVersion 3.9.0) environment using limma v. 3.40.0. Quality control was carried out by investigating raw and normalized expression intensities across arrays with Tukey box plots. Background correction and between-array quantile normalization were performed using negative/positive control probes (19) from the manufacturer with limma::neqc function (**Figure S1C**) (20–22). Next, an ExpressionSet (Biobase v. 2.44.0) object was assembled to hold assay, gene, and phenotype data (23). Principal Component Analysis (PCA) was used to inspect dataset structure and biological and technical effects. PCA was conducted with FactoMineR v. 1.41 (24) and visualized with factoextra v. 1.0.5 and cowplot v. 1.0.0 (**Figures S2A–F**). Outlier samples were removed before attempting statistical inference based on the first three principal components (**Figure S2**). Differential expression analysis was performed by fitting gene-wise linear models with moderated standard errors by the empirical Bayes method (22, 25). The final model included the independent variables: chip (categorical with seven levels), sex (categorical, two levels), treatment (categorical, two levels), and individuals as a random effect. Genes were mapped to Entrezid and HGNC official symbols using the illuminaHumanv4.db v. 1.26.0 annotation. Duplicated Entrezid were removed by keeping the one with the largest average across all arrays. Finally, nominal P-values were inspected with histograms and adjusted for multiple testing with the Benjamini-Hochberg method to control the false-discovery rate (FDR) (26). Genes were considered differentially expressed (DE) if FDR ≤ 10% and absolute fold-change ≥ 1.5 (|log_2_FC| ≈ 0.58) with alternative thresholds indicated wherever used. A volcano plot was drawn to illustrate the DE results with ggplot2 v. 3.3.0 (27). Exploratory hierarchical clustering was constructed with the top 92 DEGs using pheatmap v. 1.0.12 (28), with Euclidean distance (samples) plus average agglomeration and Pearson correlation (genes). Raw and normalized data are available in the Gene Expression Omnibus (GEO) accession GSE162416. Also, data and computer source code are readily available in Zenodo (https://dx.doi.org/10.5281/zenodo.4401968).

### Over-Representation Analysis (ORA) and Gene Set Enrichment Analysis (GSEA)

Gene ontology (GO) biological processes (BP) were evaluated separately for up- and downregulated DE lists using clusterProfiler v. 3.12.0 (29), fgsea v. 1.10.0 (30) and org.Hs.eg.db v.3.8.2 annotations. The universe set contained all Entrezid genes (n = 21207) used in DE analyses. The minimum gene set size was 5 and the FDR cutoff was set at 10%. Enriched BP were visualized with dot plots or heat plots. For GSEA (**Figure S3**), the gene list was constructed using limma’s estimated log_2_FC. GO BP and Reactome GSEA were estimated with 5000 permutations and with gene sets containing at least five genes (29, 31, 32).

### Comparison to Infected Schwann Cell Dataset

Microarray dataset GSE35423 was processed as described elsewhere (12, 14). DEGs from Schwann cells infected with *M. leprae* for 48 hours were filtered by FDR ≤ 10% and |log_2_FC| ≥ 0.26 (20% difference). Genes differentially expressed common to both datasets were visually compared with a dot plot along with log_2_FC and confidence intervals from original results (ggplot2 v. 3.3.0). Since the number of common DEG was large, only the top 50 DEG (sorted by decreasing log_2_FC) with same and opposite modulation signs were drawn. **Table S6** contains the full results. UpSetR v.1.4.0 was used to visualize the intersection between DEG according to the dataset and modulation sign.

### Gene Set Variation Analysis (GSVA)

Pathway activity was estimated using GSVA with custom gene sets (33). GSVA is an unsupervised non-parametric alternative to ORA and GSEA as it does not depend on prior selection or ranking of the genes from group comparisons. The score produced by GSVA can be interpreted as the coordinated activation of genes from the gene set, summarizing the expression profile within individual samples. The gene sets used herein were compiled from literature and functional annotation databases. The ‘granulomatosis’ gene set was assembled with genes sourced from the Human Phenotype Ontology (HP:0002955), Gene Ontology (GO:0002432), and DisGeneNet (granulomatous diseases with score ≥ 0.1) totaling 30 genes (**Table S7**). The macrophage polarization signatures are mainly from (34), where ‘M1’ (n genes = 25), ‘M2’ (n=20), ‘M2a’ (n=12), ‘M2b’ (n=9) and ‘M2c’ (n=12). ‘Pro-M1’ and ‘Pro-M2’ gene sets were built from literature (35, 36). Autophagy genes were retrieved from Reactome v.75 accessions: ‘Macroautophagy’ (R-HSA-1632852.8, n=137), ‘Chaperone Mediated Autophagy’ (R-HSA-9613829.3, n=22), and ‘Late endosomal microautophagy’ (R-HSA-9615710.3, n=34). Wilcoxon signed-rank test was used to test the differences between mean ranks between infected and mock macrophages signatures. Spearman’s rank correlation coefficient was calculated alongside 95% confidence intervals using DescTools R package v.0.99.40 (37). Principal component analysis (PCA) was applied to further explore the macroautophagy genes in the dataset. Mean-centered and variance standardized expression matrix with the 137 genes from macroautophagy gene set was subjected to PCA computed with FactoMineR v.2.4 (24). PCA scatter plot and contribution bar plots were graphed with factoextra v.1.0.6.

## Results

### Differentially Expressed Genes in MDMs Infected With *M. leprae*

Here, we included 29 healthy volunteers (15 females and 14 males) with an age range of 20-30 years. PBMCs were collected and used to obtain monocytes for differentiation to MDMs, which were subsequently infected with *M. lepra*e at 10:1 MOI for 48 h. Microarray quality control was used to discard aberrant arrays, and outlier samples were removed (**Figure S2**).

**Figure 1:**
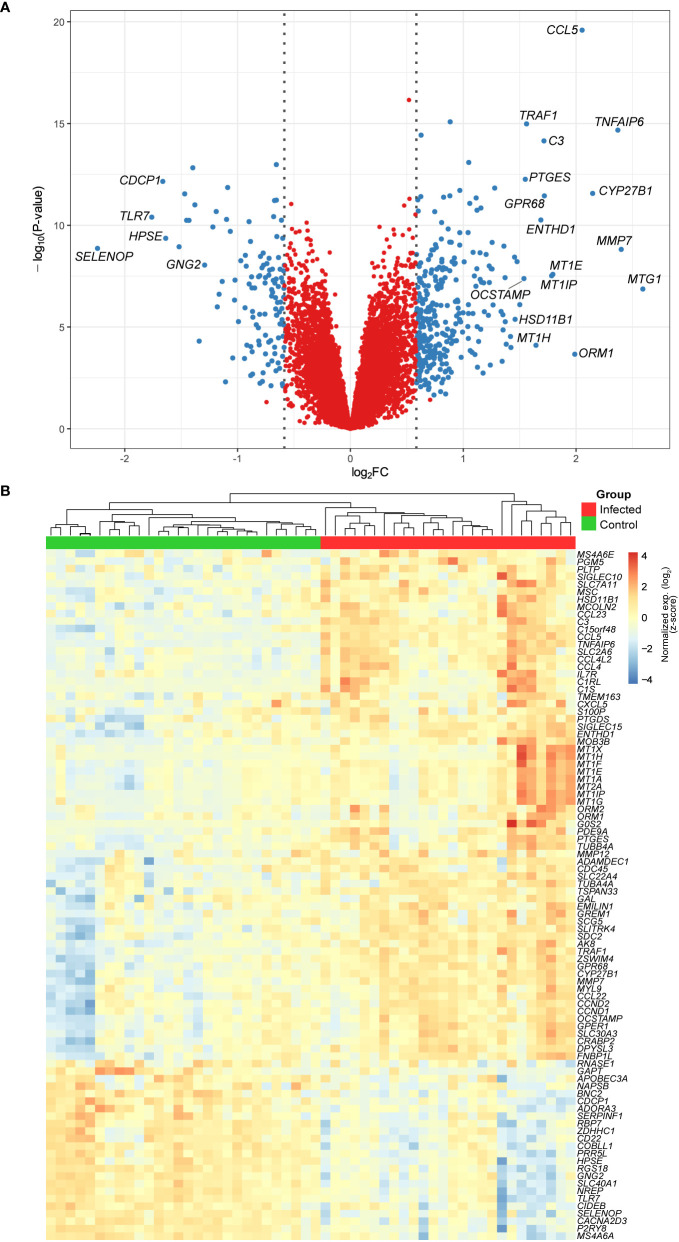
**(A)** Volcano plot showing the DEGs from monocyte-derived macrophages infected with live *M. leprae* (MOI 10:1) for 48 h. Blue dots represent genes with an FDR ≤ 10% and |log_2_FC| ≥ 0.58. Gene symbols are given for those with an FDR ≤ 10% and |log_2_FC| ≥ 1.5. **(B)** Heatmap and unsupervised hierarchical clustering of genes with an FDR ≤ 10% and |log_2_FC| ≥ 1 (n = 92). Samples were clustered based on Euclidean distance and genes with Pearson correlation coefficient, both with average agglomeration. Color key displays expression values in standard deviation units away from the mean (i.e., scaled and centered row-wise). FDR, false discovery rate; FC, fold change.

We designed our study to evaluate early changes in the macrophage-*M. leprae* interaction. Thus, human MDMs were analyzed after 48 h of infection with *M. leprae*, which resulted in 325 unique upregulated (FDR ≤ 10% and log_2_FC ≥ 0.58) and 117 downregulated (FDR ≤ 10% and log_2_FC ≤ -0.58) genes. Genes with the largest effect size (|log_2_FC| > 1.5) are annotated in the volcano plot of **Figure 1A**, of which some members of the metallothionein family can be observed, like *MT1G*, *MT1E* and *MT1P*. **Figure 1B** shows a heatmap with hierarchical clustering of all samples and the top 92 genes with an FDR ≤ 10% and |log_2_FC| ≥ 1. The heatmap demonstrates a cluster pattern that clearly distinguishes infected from non-infected samples (**Figure 1B**), at the same time highlighting the high heterogeneity among individuals for some genes, such as *ORM1*, *MT1G*, *MT1H*, *MMP12*, *CXCL5*, *COL22A1*, and *GAL*. Some of the gene families identified such as metallothioneins, chemokines, and interleukins are involved in inflammation and autophagy, and among the induced genes, we found that *MT1G*, *MMP7*, *TNFAIP6*, *CYP27B1*, and *CCL5*/*RANTES* had the largest fold-changes (**Figure 1A** and **Table S1**). Conversely, among the most repressed were *SELENOP*, *TLR7*, *CDCP1*, *HPSE*, and *GNG2* (**Figure 1A** and **Table S1**).

**Figure 2:**
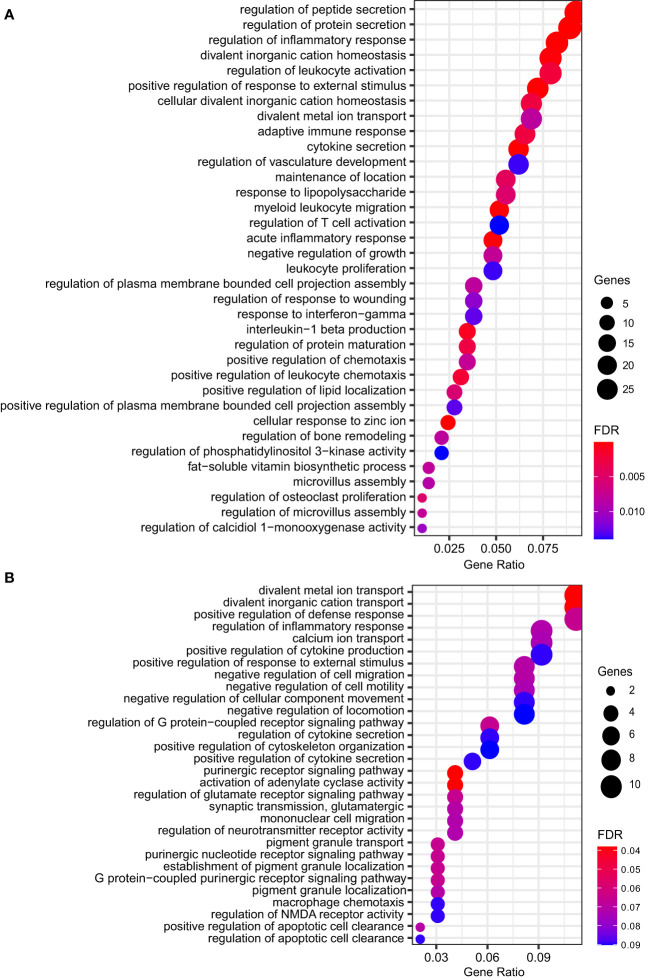
Dot plot showing the top significant GO biological processes enriched from ORA of genes **(A)** upregulated (n = 35) or **(B)** repressed (n = 30) by *M. leprae* infection. Gene ratio is the fraction of genes belonging to an ontology over the total number of modulated genes. Circle size shows the number of modulated genes per biological process. FDR, false discovery rate.

To further understand which biological processes the DEGs were involved in, we performed an over-representation analysis (ORA) with Gene Ontology Biological Process annotation. ORA revealed several biological processes modulated by *M. leprae* infection, whereby the expression patterns of the MDM genes were consistent with resistant responses and a reprogramming profile that would induce *M. leprae* killing and infection control. Strikingly, the upregulated pathways had a greater number of DEGs and more robust FDR. Among these predominantly induced pathways, there were redundancies, which are expected since several of these genes participate in multiple pathways. Nevertheless, there is a clear activation of ‘regulation of inflammatory response’, ‘regulation of leukocyte activation’, ‘cytokine secretion’, ‘regulation of T cell activation’, ‘response to IFN-gamma’, as well as some others (**Figure 2A** and **Table S2**). On the contrary, downregulated genes pertain to biological processes such as membrane lipid metabolic process, sphingolipid metabolic process, and locomotory behavior (**Figure 2B** and **Table S3**).

### Commonly Modulated Genes in Macrophages and Schwann Cells Upon Infection With Live-*M. leprae*

**Figure 3:**
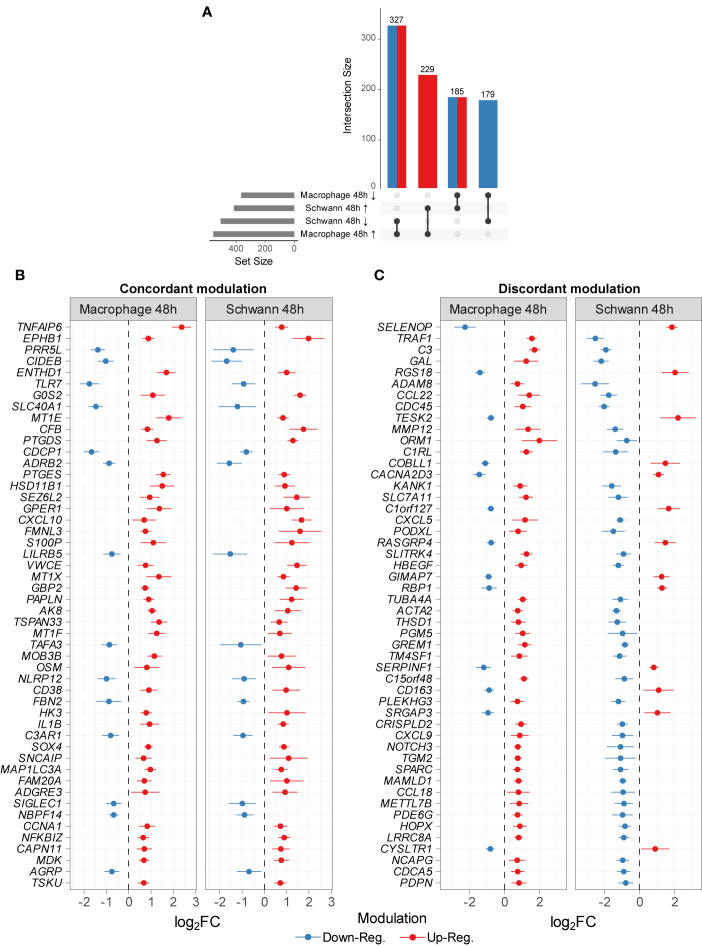
Common DEGs between this study and Schwann cells infected with *M. leprae* (MOI 1:100 for 48 h) [GSE35423] (12). **(A)** The number of commonly DEG by dataset and modulation sign passing |log_2_FC| ≥ 0.26 and FDR ≤ 10% in both datasets. Arrows indicate (↑) upregulation and (↓) downregulation. Top 50 DEG common to both datasets modulated in the same manner **(B)** or oppositely **(C)**. Points represent log_2_FC (unstandardized) from each dataset original differential expression analysis alongside error lines indicating nominal 95% confidence intervals. All genes shown have an FDR ≤ 10% and a difference in mean expression of at least 56% (≈ 0.65 log_2_FC).

As a comparison, we decided to use a public dataset from *M. leprae* infected (100:1) Schwann cells where approximately 30-100 intracellular bacilli per cell were observed (2). In this case, a shift toward a de-differentiation phenotype was noticed. By comparing the lists of DEGs from this study and the results from Schwann cells infected with live *M. leprae* (100:1) for 48 h, we identified 920 DEGs with FDR ≤ 10% and a difference in mean expression of at least 20% (i.e., |log_2_FC| ≥ 0.26). Of these, 229 (24.89%) were upregulated in both datasets, while 179 (19.45%) were jointly downregulated (**Figure 3A** and **Table S6**). On the other hand, 512 genes (55.65%) were regulated in opposite directions in the two experiments (**Figure 3A**). We next graphed the top 50 genes with the greatest |log_2_FC| for both concordant and discordant DEG, as this can be used to further enlighten both similarities and differences in how the pathogen interacts with each host cell as well as the effect of a higher/lower multiplicity of infection (**Table S6**). Indeed, a higher multiplicity of infection (Schwann dataset) leads to activation of the type I IFN pathways (*OAS1, TRIM7, TNFSF10*) and subversion of energetic metabolism where increased glucose uptake is redirected from glycolysis/mitochondrial respiration to lipid metabolism (*MVK, DHCR7, HMGCS1, LDLR, MSMO1*)  (3, 12). In the same manner, some members of TNF signaling, inflammasome pathway, and regulators of NFκB and JNK pathways were upregulated in MDMs but repressed in Schwann cells, such as *TNF*, *CXCL5*, *FAS*, *MAPK13, TRAF1, NKFB2, AIM2*, and* PANX1 *(**Figure 3C** and **Table S6**). Furthermore, in Schwann cells, we observed a consistent upregulation of genes involved with prostaglandin biosynthesis, lipogenesis, mitochondrial metabolism, and negative regulation of immune *ADORA1*, *LDLR*, *NOV*, *PPARA*, *SERPINF1*, *FFAR4, *and* ARG2)*. The patterns were quite distinct from the MDMs where Schwann cells showed a more pronounced expression of genes *SELENOP*, *GAL*, *RGS18, AIF1, DEF6*, *ANGPTL6, DHCR7*, *MVK*, and *MSMO*, some involved in cholesterol biosynthesis, along with *LACC1/FAMIN*, which is also genetically associated with leprosy (**Table S6**).

### Infected MDMs Express Mainly M1 Polarization Genes That Are Correlated With Granuloma Formation

**Figure 4:**
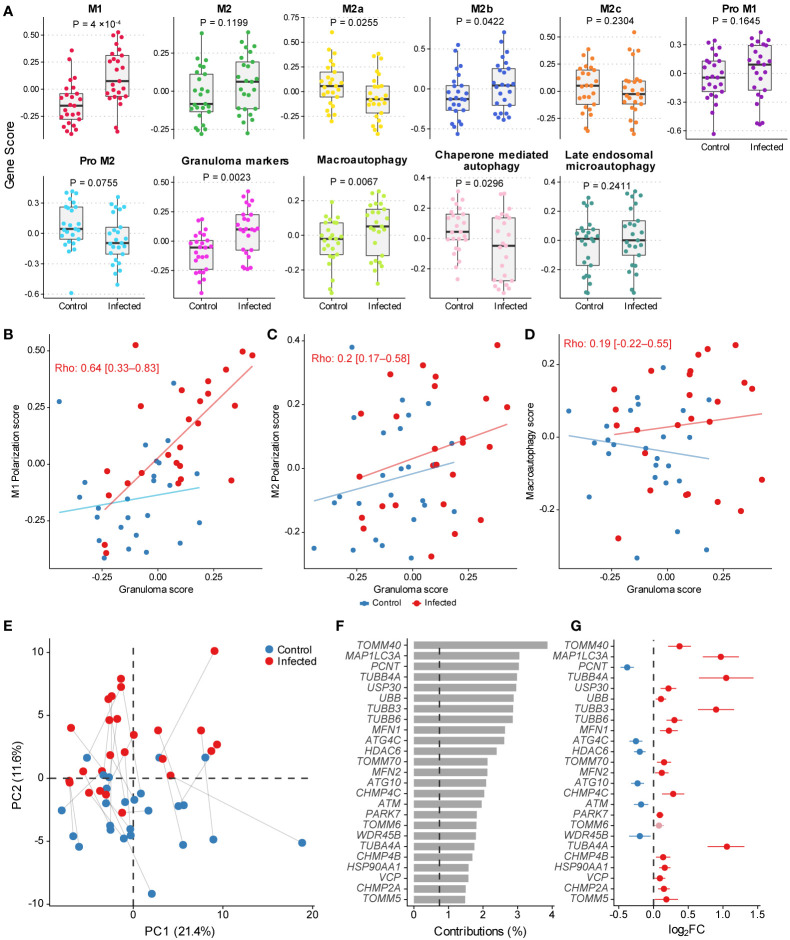
**(A) **Gene scores for macrophage polarization profiles, granulomatosis and autophagy. Tukey box plots display first, second (median), and third quartiles with whiskers extending ± 1.5 × interquartile range (IQR). Each point illustrates a MDM culture (n=50) from one human donor in mock and infected conditions (paired within donor). Nominal P-values displayed are from Wilcoxon signed-rank test. **(B–D) **Scatter plots illustrating the correlation between granulomatosis, M1, M2, and macroautophagy gene scores. Spearman’s rank correlation coefficient is shown with nominal 95% confidence intervals calculated only with the infected condition (n=25). Slope and intercept for drawing the lines were estimated using robust linear regression from the MASS v.7.3-53.1 R package. **(E) **Scatter plot with the two first principal components from PCA on the subset of 137 macroautophagy-related genes. Lines connect samples from the same donor. **(F) **Percentage contribution of the top 50 genes most correlated to PC2. **(G) **Log_2_FC estimates from differential expression analysis for the top 50 macroautophagy-related genes from PCA and their nominal 95% confidence intervals. Light-shaded red indicates gene with FDR > 10%.

The somewhat contrasting gene expression profiles identified before suggested that the infected MDMs could be expressing M1 markers with autophagic and phagocytic profiles. To examine this, we calculated gene scores representative of multiple macrophage polarization phenotypes, autophagy, and granulomatosis, which are features often observed in paucibacillary leprosy patients. **Figure 4A** shows the scores for each signature indicating a transcriptional activity increase in genes involved with M1 macrophage polarization, as well as decreased or similar activity for M2, M2a, and Pro-M2. It seems that although genes responsible for inducing M2 phenotype are unaffected, the M2b signature shows activation upon infection, which could indicate either a mixed MDM specialization phenotype or distinctive polarization patterns between individuals, like the clinical disease presentation. As for the autophagy signatures, macroautophagy is specifically active in infected MDMs, whereas chaperone-mediated autophagy appears the opposite (**Figure 4A**). Finally, we tested the correlation between granulomatosis gene scores with M1, M2, and macroautophagy scores. There was a moderated positive correlation between M1 polarization signature with granulomatosis, and no correlation with M2 score, which together corroborated our hypothesis that a pro-inflammatory macrophage profile seems predominant in this model with low MOI (**Figures 4B, C**). The granulomatosis signature does not correlate with macroautophagy, which could indicate that these processes may not be simultaneously active at the transcriptional level (**Figure 4D**). Finally, we wanted to see which macroautophagy genes were driving the signature activity signal. Principal component analysis (PCA) with the 137 genes from the macroautophagy signature revealed that the second principal component (PC2) separated MDMs according to the infection treatment, explaining around 12% of the variability (**Figure 4E**). As expected, there is considerable between-individual variation and noise captured mainly by the first and second principal components (PCs). We then explored PC1, and only the “microarray chip” variable partially correlated with this PC (data not shown). Some uninfected MDMs had basal higher expression (placed vertically higher across PC2) as seen in blue dots over the horizontal dashed line marking zero (**Figure 4E**). Next, we extracted the top 25 genes most correlating to PC2. The gene *TOMM40 *alone contributed with explaining almost 4% of the variability of that axis (**Figure 4F**), where more than 25 genes contribute with more variability than would be expected if contributions were uniform (dashed vertical line in **Figure 4F**). Infected MDMs separated from their mock pair by great distance vertically (**Figure 4F**) are cells with the most difference in expression for the genes correlating with PC2, which varies among individuals. Most of the top genes with higher contributions to PC2 showed upregulation after infection with live *M. leprae, *with some exceptions that were significantly downregulated (**Figure 4G**).

## Discussion

Here, we evaluated the gene expression patterns of *M. leprae*-infected MDMs, at a 10:1 MOI for 48 h, using a large-scale technique. The data were consistent with an M1-like differentiation profile, with several enriched pathways associated with increased microbicidal activity, T cell activation, and IFN-gamma cytokine secretion. Among the most DE genes were those found in the vitamin D processing pathway (*CYP27B1*, *VDR*, *IL1B*), CC-chemokine ligands (*CCL5, CCL4, CCL3, CCL4L2, CCL2*), receptors involved in cellular migration (*GREM1*, *MCOLN2*, *MDK*, *CCL3*), and inflammation (*CCL5*/*RANTES*). It seems that a combination of MDM differentiation with *M. leprae* infection at a low multiplicity of infection (10:1) induces a gene expression program in agreement with a protective response. One of these genes, *CCL5/RANTES*, is a key chemoattractant for monocytes in the skin suggesting a pro-inflammatory feedback loop during *M. leprae* infection toward M1-like macrophages, which is consistent with the higher expression in PB leprosy (38, 39). Curiously, the chemokine-clustered genomic region has been associated with leprosy indicating that genetic variations within this gene could contribute to clinical form polarization (40). We observed an upregulation of metallothionein gene’s expression, like *MT1E* and *MT1G*. These genes produce proteins that regulate metal availability, while zinc homeostasis is crucial to activation of transcriptional factors and reactive oxygen species and have been associated with antimicrobial immune responses (41).

Some metalloproteinases, such as *MMP7*, *ADAMDEC1* and *MMP12*, were also highly differentially expressed. These are involved with tissue/matrix remodeling and host defense. It is interesting that the macrophages in this model appear to have a regulatory activation of gene expression involved in tissue remodeling. Indeed, *MMP2* and *MMP9* expression and activity were higher among T-pole patients (42). This pattern is dependable with the reshaping of cellular morphology and the adjacent tissue during M1-like macrophage differentiation observed here.

The upregulation of mitochondrial genes in *M. leprae*-infected-macrophages, as compared to Schwann cells, is also consistent with the aforementioned alterations observed in infected MDMs toward epithelioid transformation programming. In this same direction, *NDUFAF6*, which encodes a protein that participates in the mitochondrial respiratory chain complex I (NADH:ubiquinone oxidoreductase) assembly; *TACO1*, which is involved in the translational activation of mitochondria cytochrome c; and *TOMM40*, the encoded protein of which produces a pore to channel protein precursors into mitochondria were also observed. We also detected an over-expression in MDMs of metabolite transporters across the inner mitochondrial membrane (*SLC25A12*).

Other sets of induced genes in the MDMs were involved in tubulin assembly and cytoskeleton remodeling, such as *TUBB6*. Some of these genes are involved in autophagy and the mTOR pathway (43), which is increased during macrophage differentiation in the presence of a low *M. leprae* MOI or dead mycobacteria (44). Curiously, Yang and colleagues showed that a 20:1 MOI leads to higher CD163 and an anti-inflammatory profile with higher IL-10 and lower HLA expression, although dead *M. leprae* induced proinflammatory cytokines (45). In clinical samples these patterns are observed in low bacterial index patients (T-pole) and type 1 reactional patients (RR) where vitamin D-mediated microbicidal clearance is observed. Furthermore, IFN-gamma and IL-15 have been demonstrated to gear the polarization of M1-like macrophages, exhibiting a phenotype that is probably orchestrating the milieu inducing granuloma formation and autophagy (44, 46). Thus, there is a clear antagonistic pattern that, on the one hand, we observed granuloma formation and M1-like polarization for macrophages (10:1 MOI), whereas lipid biogenesis, wound healing and a phagocytic signature were observed for Schwann cells (100:1 MOI). This M2-like profile occurred in both Schwann cells and macrophages when higher concentrations of *M. leprae* were used to infect the cells (11, 12, 47). Indeed, molecular and biochemical subversion induced by *M. leprae* infection leads to a type I IFN response reducing autophagy (12, 48) and turning on the Warburg-like effect (3). This is expected since this downregulation was detected as associated with diminished capacity to produce ATP through the respiratory chain (3) and the redirection of glycolysis to lipid biosynthesis. Furthermore, we observed a prominent upregulation toward higher MOI (100:1) when genes associated with cholesterol and fatty acid metabolism (*DHCR7*, *MVK*, and *MSMO*, and *LACC1*/*FAMIN* were analyzed, which are pathways involved in lepromatous leprosy immunopathogenesis.

Genes associated with leprosy outcome are also involved with ulcerative colitis pathogenesis and other diseases, such as Crohn’s disease, Parkinson’s and Alzheimer’s (49). Curiously, some of the genes highlighted here, such as *TOMM40* and *TUBB6*, were associated not only with neurodegenerative diseases, like Alzheimer’s, but also with ulcerative colitis (50, 51). These proteins interact with *LRRK2*, which is a gene that has been independently associated with leprosy, and Parkinson’s disease (49, 52). Another important gene upregulated in *M. leprae*-Schwann cells was *LACC1.* The protein which *LACC1/FAMIN* encodes is involved in fatty acid oxidation and bacterial clearance. Interestingly, the gene was associated with leprosy in several studies (53, 54).

The global gene expression can also be analyzed in a genotype-phenotype correlation perspective. In leprosy, whole blood cells stimulated with *M. leprae* sonicates indicate quantitative trait loci (eQTL) associated with transcript levels when samples were compared before and after stimulation. The data revealed SNPs controlling immunoinflammatory responses such as type II IFNs and bacterial/pathogen recognition (55). Indeed, there are SNPs regulating expression of genes such as *LACC1*, which indicates that it is likely that a combination of *M. leprae*-induced activation with genetic polymorphisms may define the commitment toward the T- or L-pole. Thus, together these could contribute to identifying potential pharmacologic targets as adjuvants to personalize treatment for each specific clinical form.

## Data Availability Statement

The datasets presented in this study can be found in online repositories. The names of the repository/repositories and accession number(s) can be found below: https://www.ncbi.nlm.nih.gov/geo/, GSE162416.

## Ethics Statement

The studies involving human participants were reviewed and approved by Local Ethics Committee, Lauro de Souza Lima Institute. The patients/participants provided their written informed consent to participate in this study. The animal study was reviewed and approved by Local Ethics Committee, Lauro de Souza Lima Institute.

## Author Contributions

AP, MM, and VB contributed to the design and implementation of the research. TL-C, AL, and MM to the writing of the manuscript. BM, DB, RC, and GG performed the experiments and processed the experimental data. TL-C performed the analysis. PR and DB contributed to *M. leprae* maintenance in athymic nude mice and purification. TL-C, MM, and AP to the analysis of the results. All authors provided critical feedback and helped shape the research, analysis and manuscript. All authors contributed to the article and approved the submitted version.

## Funding

This study was funded in part by the Fundação de Amparo do Estado do Rio de Janeiro (FAPERJ, E_09/2019 - Cientista do Nosso Estado – 2019 and E_34/2014 - PENSA RIO – Apoio ao Estudo de Temas Relevantes e Estratégicos para o RJ - 2014) and the Conselho Nacional de Desenvolvimento Científico e Tecnológico (CNPq) [grant #313657/2018-1 and 4000170/2017-2]. Fundação de Amparo à Pesquisa do Estado de São Paulo - FAPESP, Grant no. 2015/01744-9.

## Conflict of Interest

The authors declare that the research was conducted in the absence of any commercial or financial relationships that could be construed as a potential conflict of interest.
