# Erratum: Large-Scale Gene Expression Signatures Reveal a Microbicidal Pattern of Activation in *Mycobacterium leprae*-Infected Monocyte-Derived Macrophages With Low Multiplicity of Infection

**DOI:** 10.3389/fimmu.2022.852579

**Published:** 2022-02-03

**Authors:** Frontiers Production Office

**Affiliations:** Frontiers Media SA, Lausanne, Switzerland

**Keywords:** macrophages, *Mycobacterium leprae*, eQTLs, SNPs, host-directed therapy, leprosy, tuberculosis

Due to a production error, there was a mistake in **Figure 2** as published. The filled circles in the dot plot did not export correctly. The corrected **Figure 2** appears below. The publisher apologizes for this mistake.

**Figure 2:**
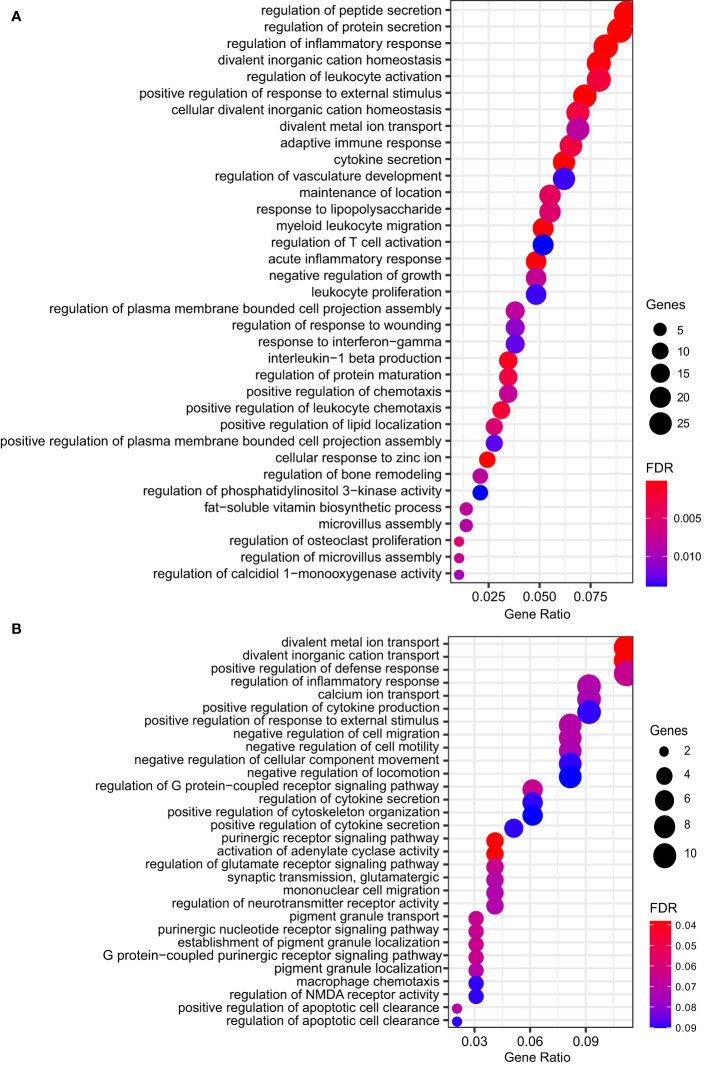
Dot plot showing the top significant GO biological processes enriched from ORA of genes **(A)** upregulated (n = 35) or **(B)** repressed (n = 30) by *M. leprae* infection. Gene ratio is the fraction of genes belonging to an ontology over the total number of modulated genes. Circle size shows the number of modulated genes per biological process. FDR, false discovery rate.

The original version of this article has been updated.

