# Supplementary material for: Large-Scale Gene Expression Signatures Reveal a Microbicidal Pattern of Activation in Mycobacterium leprae-Infected Monocyte-Derived Macrophages With Low Multiplicity of Infection
Source: Front Immunol. 2021 Apr 16;12:647832. doi: 10.3389/fimmu.2021.647832 (PMC8085500; doi:10.3389/fimmu.2021.647832)
Supplement: Supplementary file 1 [file DataSheet_1.docx]

Supplementary Material

# Supplementary Figures and Tables

## Supplementary Figures


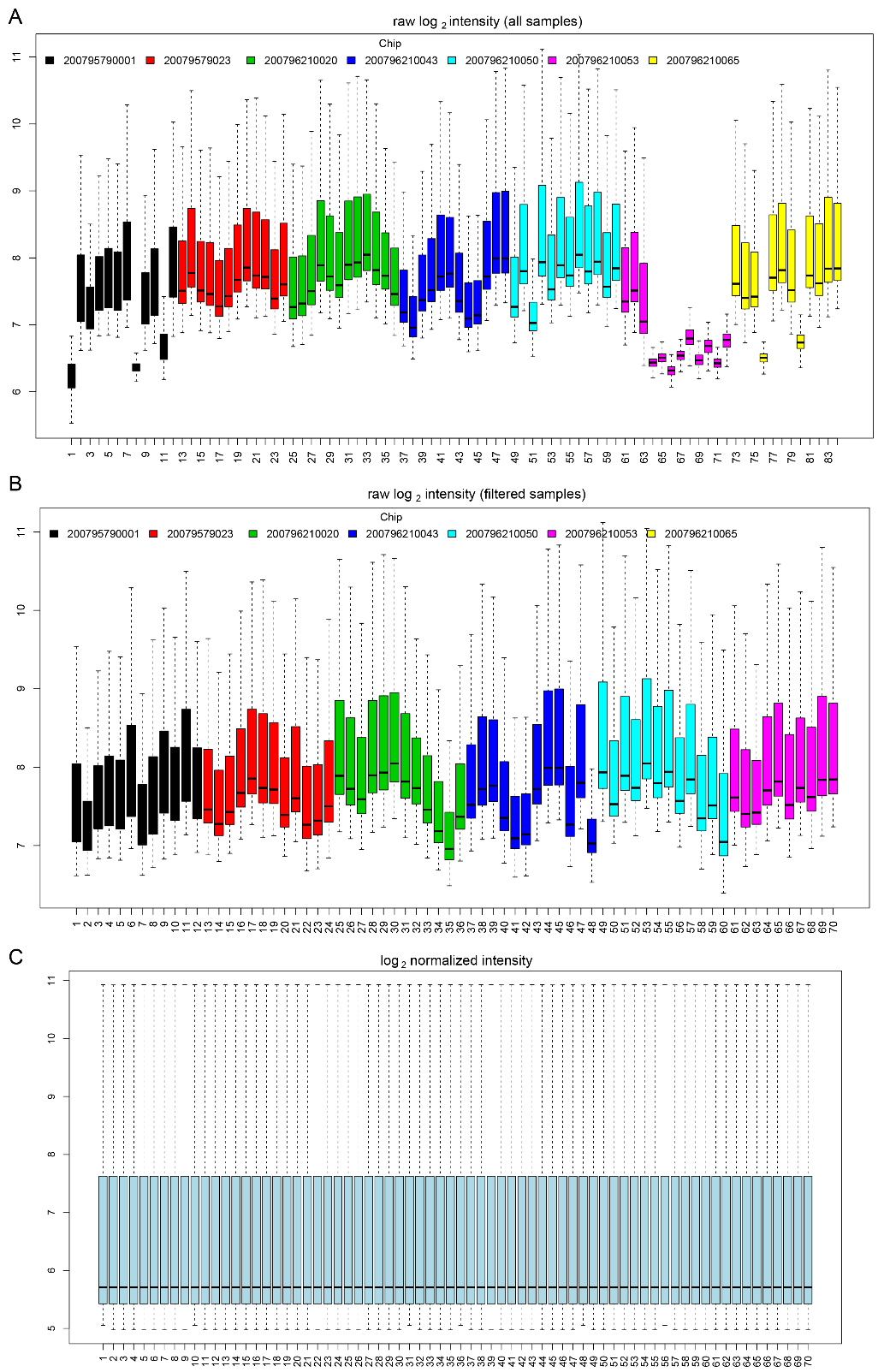


**Supplementary Figure 1.** Tukey Box plots showing array distributions: **(A)** before any procedure for all arrays, **(B)** after excluding aberrant and blank arrays, and **(C)** after background correction and normalization. Box plots show median, first and third quartiles with whiskers extending ±1.5 ✕ interquartile range (IQR) from box edges. Outlier genes have been omitted for appearance.


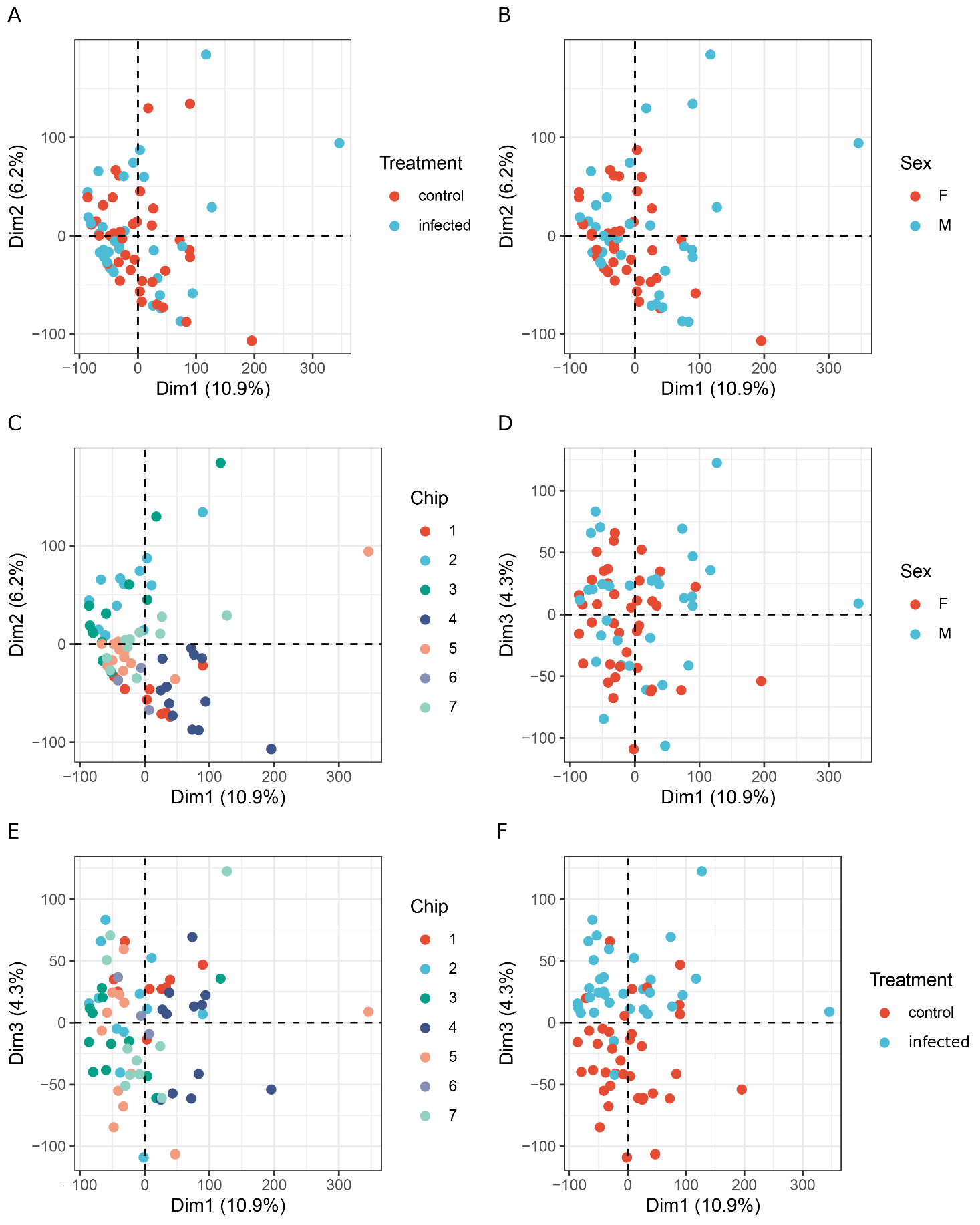


**Supplementary Figure 2.** Scatter plots of principal components from the PCA. The two first PCs are shown with coloring according to **(A)** treatment variable, **(B)** individual biological sex, and **(C)** microarray chip. Scatter plots of PC1 and PC3 with color labels for biological sex **(D)**, microarray chip **(E)**, and treatment variable **(F)**.


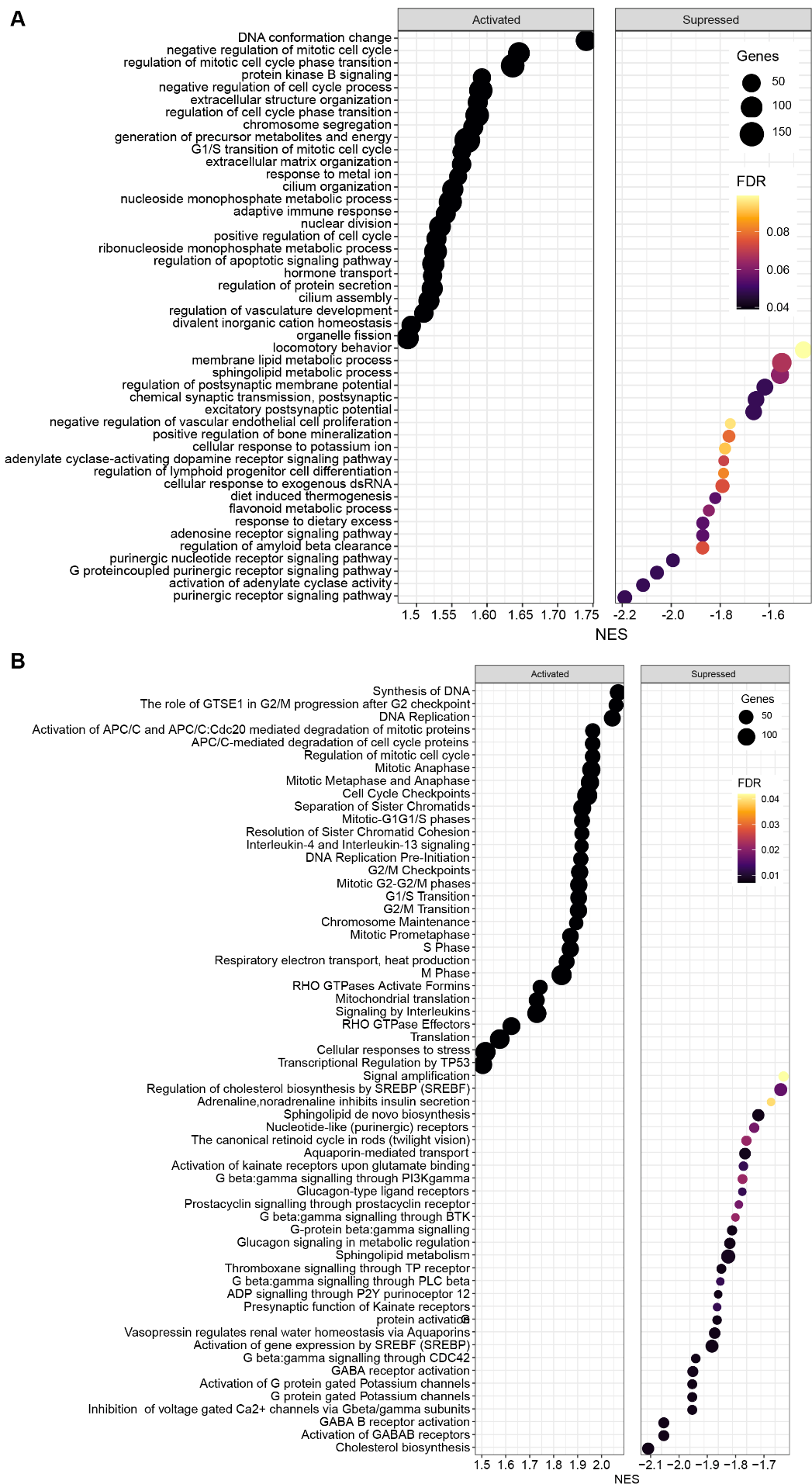
**Supplementary Figure 3.** Gene set enrichment analysis (GSEA) results of transcriptional modulation induced upon infection with live *M. leprae*. **(A)** Significant ontologies according to Gene Ontology (GO) Biological Processes (BP) annotation and **(B)** Reactome pathways. Dot plots are colored according to Benjamini-Hochberg false-discovery rate (FDR) and dot sizes are proportional to the number of genes for a given ontology/pathway. In x-axis are normalized enriched scores (NES) from GSEA.


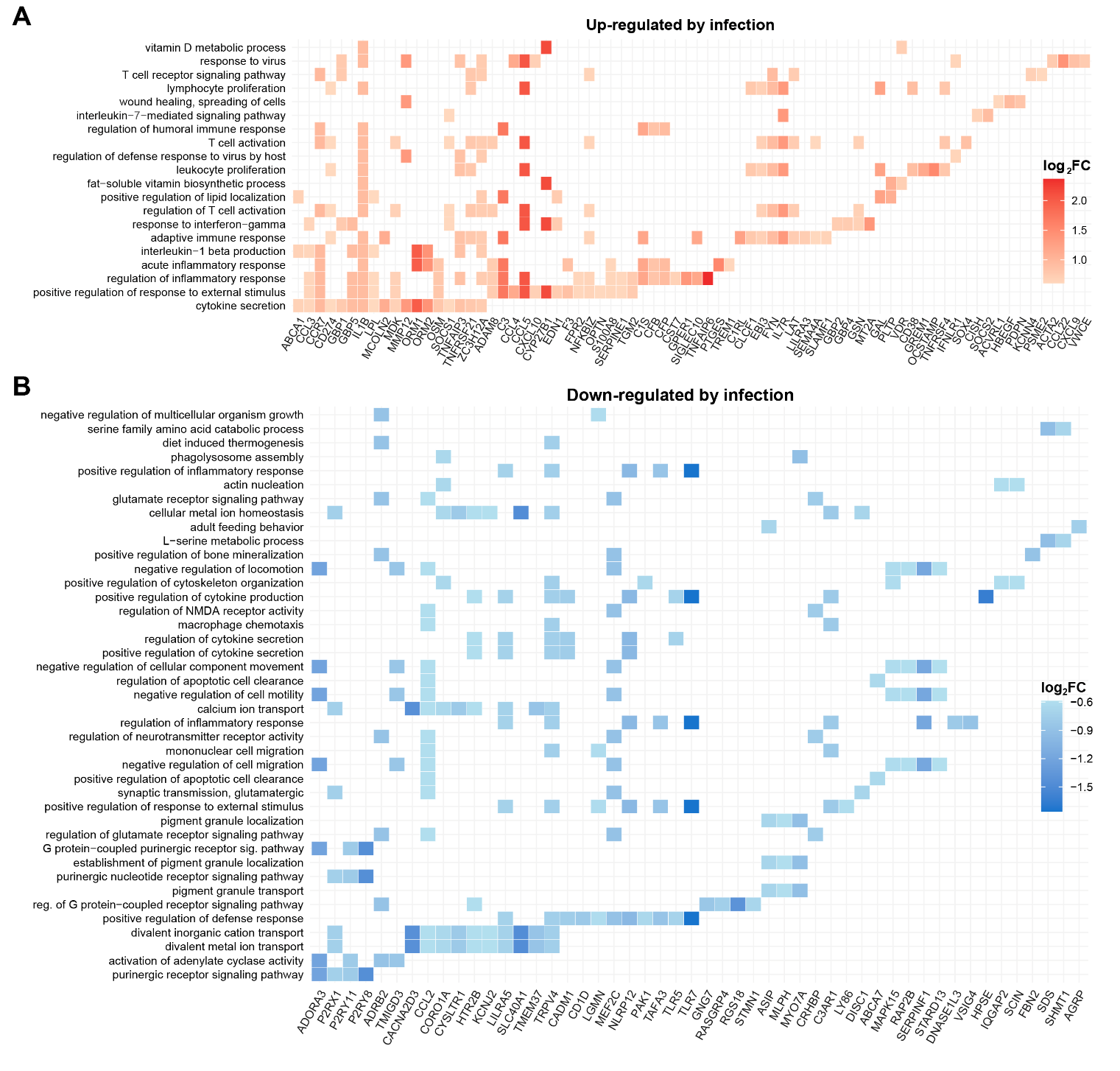


**Supplementary Figure 4.** Heat plots showing differentially expressed genes (DEG) according to its Gene Ontology biological process annotation. Up-regulated **(A)** and **(B)** down-regulated genes from selected biological processes.

## Supplementary Tables

Tables are in .XlSX format and are not included here due to their size.

**Supplementary Table 1.** Differentially expressed genes (DEGs) from monocyte-derived macrophages infected with live *M. leprae* (MOI 1:10) for 48h. Genes were considered DE when the FDR ≤ 10% and |log_2_FC| ≥ -0.58. HGNC symbols were used along with Entrezid according to illuminaHumanv4.db v. 1.26.0 annotation (Bioconductor). Genes are sorted with increasing false discovery rate (FDR).

**Supplementary Table 2.** Enriched gene ontology (GO) biological processes (BP) from genes up-regulated by infection with adjusted P-values (FDR) ≤ 10%.

**Supplementary Table 3.** Enriched gene ontology (GO) biological processes (BP) from genes down-regulated by infection with adjusted P-values (FDR) ≤ 10%.

**Supplementary Table 4.** Significant gene set enrichment analysis (GSEA) results with gene ontology (GO) biological processes (BP).

**Supplementary Table 5.** Significant gene set enrichment analysis (GSEA) results with Reactome pathways/annotations.

**Supplementary Table 6.** Differentially expressed genes in common to this study macrophage dataset and previous results from Schwann cells.

**Supplementary Table 7.** Genes used in macrophage polarization, autophagy and granuloma signatures.
